# Recent Advances in Antibody Therapeutics

**DOI:** 10.3390/ijms23073690

**Published:** 2022-03-28

**Authors:** Yong-Seok Heo

**Affiliations:** Department of Chemistry, Konkuk University, 120 Neungdong-ro, Gwangjin-gu, Seoul 05029, Korea; ysheo@konkuk.ac.kr; Tel.: +82-2-450-3408; Fax: +82-2-3436-5382

Antibody-based therapeutics have achieved unprecedented success in treating various diseases, including cancers, immune disorders, and infectious diseases. Since the approval of OKT3 in 1986, more than 100 antibody-based drugs have been approved by the FDA. In this Special Issue, “Recent Advances in Antibody Therapeutics”, promising discoveries and developments of this most prevalent biologics are presented. In addition, the review articles covering the clinical and technical advances for antibody usage and discovery could provide valuable insight into applications by clinicians and scientists in this field.

As an antibody discovery is advanced to preclinical development, it is essential to consider its biophysical properties to determine whether it can be successfully developed into an efficacious drug. Chae et al. reported the successful result for improving biophysical properties of an antibody against the L1 cell adhesion molecule for cancer therapy [1]. In this study, one of the variants derived by a computer-aided method demonstrated reduced aggregation propensity, increased stability, higher purification yield, lower pI, higher affinity, and greater in vivo anti-tumor efficacy, leading to a promising candidate antibody for preclinical development.

T-cell immune responses are initiated by the interaction between the T-cell receptor (TCR) and peptide-HLA complex. In January 2022, the U.S. Food and Drug Administration (FDA) approved KIMMTRAK (tebentafusp-tebn), which is the first T-cell receptor (TCR) therapeutics fused with an anti-CD3 T-cell engaging scFv. The approval of this biologic verified TCR-based protein engineering as a valuable therapeutic modality. Lee et al. reported a TCR-like antibody specific for HLA-A*02:01 in complex with a peptide derived from human cytomegalovirus (CMV) pp65 protein [2]. The binding affinity was matured by sequential mutagenesis of complementarity-determining regions using yeast surface display technology for its application to the diagnostics and therapeutics of CMV infection.

The FDA has approved several monoclonal antibodies neutralizing the SARS-CoV-2 virus for treating COVID-19. Kim et al. discovered human monoclonal antibodies that neutralize SARS-CoV-2 through the phage display method against the receptor-binding domain (RBD) of the SARS-CoV-2 spike protein [3]. Some of them elicit cross-reactivity with the SARS-CoV spike protein and neutralize activities on pseudo-typed and authentic SARS-CoV-2 viruses, anticipating their therapeutic and diagnostic application against SARS-CoV-2.

Bispecific antibodies against two distinct antigens have been extensively explored for therapeutic purposes. Due to a biophysical bridge between two targets by simultaneous binding, bispecific antibodies can achieve specific therapeutic efficacy with multiple MoA. Yeom et al. reported a bispecific antibody targeting VEGF and DLL4, eliciting more potent anti-angiogenic activity than investigational antibodies targeting VEGF or DLL4 alone [4]. In addition, the combination of this bispecific antibody with paclitaxel or irinotecan synergistically inhibited tumor progression in xenograft models.

A significant number of the antibodies in clinical use target interleukins (ILs) or their receptors for treating chronic inflammatory diseases, including psoriasis, rheumatoid arthritis, Castleman disease, atopic dermatitis, and asthma. Park et al. discovered a single-chain antibody variable fragment (scFv) against interleukin 33 (IL-33) to block its binding to the suppressor of tumorigenicity 2 (ST2) receptor using the phage display library [5]. Downregulation of IL-33-mediated signaling has been recognized as a therapeutic strategy to prevent allergic inflammation and chronic diseases such as asthma, atopic dermatitis, and allergic rhinitis asthma. The antibody fragment in this study can be further engineered and improved for therapeutic application.

Abe et al. recently reviewed the therapeutic antibodies in clinical use for treating severe asthma, including omalizumab, mepolizumab, reslizumab, benralizumab, and dupilumab, describing their distinct mechanisms of action, achievements, and limitations [6]. They also emphasized the importance of biomarkers for the clinical prediction of good responders to each antibody therapy. They summarized new potential targets for asthma treatment, including thymic stromal lymphopoietin (TSLP), IL-25, IL-33, IL-13, and chemokines, with investigational antibodies against them. Further studies to fully understand the pathogenesis and clarify the effects of each antibody type in asthma endotypes will guide decision-making regarding appropriate antibody therapeutics with improved efficacy.

Donà et al. reviewed the state of the art of the therapeutic antibodies targeting human papillomavirus (HPV) oncoproteins developed so far in different formats and outlined their mechanisms of action [7]. Cervical cancer is by far the most common HPV-related disease. To date, as there is no treatment for HPV itself, an infection could cause abnormal cell changes that might lead to cancer. Given that the global burden of HPV-associated cancers is unacceptably high, antibody-based therapies can be a promising strategy for fighting HPV-associated cancers in parallel with vaccination.

Kouhi et al. reviewed the various methods currently used for antibody delivery to the central nervous system (CNS) at the preclinical stage and the underlying mechanisms of blood–brain barrier (BBB) penetration, with the description of the recent efforts to improve or modulate antibody distribution and disposition into the brain [8]. Very recently, the FDA approved the first BBB-penetrating antibody, aducanumab, for Alzheimer’s disease treatment. Further development in this field can revolutionize the treatment of diseases of the CNS.

Ji et al. reviewed the recent findings on the preclinical and clinical studies of therapeutic antibodies in atherosclerosis and their underlying mechanism of action targeting LDL and cytokines [9]. In clinical trials, many antibodies targeting PCSK9, TNFα, IL-1β, IL-6, IL-17, and IL-12/23 have shown ambivalent results, with some cases showing significant alleviation of symptoms and others experiencing adverse events such as the aggravation of cardiovascular diseases. Further studies to fully understand the exact mechanism of action of the target molecules would be very helpful in overcoming the side effects or applying the appropriate treatment.

Register et al. reviewed recent advances and case studies of bioassay development for different types of bispecific antibodies [10]. As bispecific antibodies have complicated mechanisms of action, diverse structural variations, and dual-target binding, developing a bioassay method is challenging. The detailed description of the diverse bioassay technologies and case studies in this review article can provide insight into designing strategies for developing and characterizing bispecific antibodies.

Lin et al. reviewed current findings and applications to identify functional antibodies, especially agonist antibodies capable of activating cell-signaling cascades, selected from combinatorial antibody libraries [11]. This review suggests that the use of phenotypic screening with combinatorial antibody libraries shows great promise in allowing the identification of receptor pleiotropism and the selection of antibodies capable of modulating the differentiation, growth, and function of cells.

Despite the high value of G-protein-coupled receptors (GPCRs) as a therapeutic target, only two antibodies targeting GPCR, erenumab and mogamulizumab, have been approved by the FDA. One of the main reasons for the slow development of therapeutic antibodies against this attractive drug target is the difficulty in preparing functional GPCR antigens. Ju et al. reviewed various successful technologies to prepare active GPCR antigens that enable the isolation of therapeutic antibodies to proceed toward clinical validation [12].

The treatment of bladder cancer has advanced rapidly since the approval of immune-checkpoint inhibitors. Bednova et al. reviewed recent clinical trials of targeted therapeutics, including the antibody-drug conjugates (ADCs) enfortumab vedotin and sacituzumab govitecan, for patients with metastatic bladder cancer [13]. In addition, they described the cost-effectiveness of these targeted molecular therapeutics relative to the antibody drugs blocking immune checkpoints PD-1 and PD-L1.

The five research articles and eight reviews published within this Special Issue, “Recent Advances in Antibody Therapeutics”, are excellent studies of the advances made in the field of therapeutic antibodies for treating various diseases. I want to thank all the authors and reviewers for their contributions and the editor, Sydney Tang, for outstanding dedication and professionalism throughout this Special Issue.
